# Repeated Clozapine Administration Causes Extensive Changes to the Expression of Coding and Non-coding RNAs, Including miR-124, in the Mouse Frontal Cortex

**DOI:** 10.1007/s12035-025-05199-4

**Published:** 2025-07-21

**Authors:** Rabha Mussa Younis, Dalia Y. Al Saeedy, Mikhail G. Dozmorov, Fay M. Jahr, Shravani Malay, Sina Mahdiani, Bashir Idris, Joel Castillo, Patrick M. Beardsley, Joseph L. McClay

**Affiliations:** 1 Department of Pharmacotherapy and Outcomes Science, School of Pharmacy, Virginia Commonwealth University, Richmond, VA USA; 2 Department of Biostatistics, School of Public Health, Virginia Commonwealth University, Richmond, VA USA; 3 Department of Pharmacology and Toxicology, School of Medicine, Virginia Commonwealth University, Richmond, VA USA; 4 Center for Biomarker Research & Precision Medicine, Virginia Commonwealth University School of Pharmacy, Richmond, VA USA; 5 Virginia Institute for Psychiatric and Behavioral Genetics, Virginia Commonwealth University, Richmond, VA USA

**Keywords:** Antipsychotic drugs, Schizophrenia, Brain, Non-coding RNA, RNA sequencing, Gene expression

## Abstract

Clozapine is arguably the most effective antipsychotic drug for the treatment of schizophrenia, but the mechanisms underlying its efficacy are poorly understood. Therefore, we perform deep RNA sequencing to test for differential transcription and exon use resulting from clozapine’s effects in the mouse frontal cortex, and integrate our findings with known schizophrenia risk genes. We used a dose (4 mg/kg/day, i.p.) and duration (21 days) to approximate clinical exposure, followed by a 24-h washout to determine persistent changes resulting from biological remodeling. We observed significant (FDR < 0.05) differential expression of both mRNAs and long noncoding RNAs (lncRNAs), which were enriched in RNA processing and splicing pathways. Among the most significant lncRNAs, showing 2.3-fold upregulation, was the microRNA 124 host gene (*Mir124a-1hg*), a major source of miR-124, one of the most abundant microRNAs in the brain. Quantitative PCR analysis of the mature microRNAs *miR-124-3p* and *miR-124-5p* revealed a significant dose-dependent upregulation of *miR-124-3p* following 21-day repeated clozapine administration. RNA splicing was also profoundly impacted by clozapine, as revealed by differential exon use analysis, with mouse orthologs of 50 schizophrenia risk genes from the Psychiatric Genomics Consortium among the genes affected. These genes were enriched in “apical dendrite” and “distal axon” ontologies, supporting prior evidence that clozapine may target cortical pyramidal neuron deficits implicated in schizophrenia. Overall, this study demonstrates the profound effect of clozapine on cortical gene expression, affecting abundance of splicing of coding and non-coding transcripts. Future studies are needed to fully characterize our findings as potential preclinical markers of clozapine response.

**Supplementary Information:**

The online version contains supplementary material available at 10.1007/s12035-025-05199-4.

## Introduction

Second-generation (atypical) antipsychotic drugs, with the exception of clozapine, are first-line treatments for acute and maintenance therapy of schizophrenia (SCZ) [1, 2]. Clozapine is not used as a first-line treatment for SCZ because patients require continuous safety monitoring due to a rare but potentially lethal side effect (agranulocytosis). However, among the current drugs, clozapine is considered one of the most effective [3, 4] and shows clear superiority over other antipsychotics for treatment-resistant patients [5, 6]. Approximately 30–40% of SCZ patients are considered to be resistant to antipsychotic therapy, where resistance is usually defined as failure to respond to two or more different antipsychotics [7]. If future antipsychotics could be developed with the efficacy of clozapine without its dangerous and deleterious side effects, these could be superior treatments for SCZ [4]. However, the mechanisms underlying clozapine’s superior efficacy are not well understood. Therefore, in this study, we conduct a broad investigation of the genomic effects of repeated clozapine administration in the mouse frontal cortex.

There is growing recognition for the role of genomic regulation in antipsychotic response [8, 9], and gene expression studies have previously been used in an attempt to better understand the molecular mechanisms of antipsychotic drug response, including clozapine response [10]. Mechanistic studies of clozapine’s effects focused on humans have typically used postmortem brain tissue [11], but postmortem tissue can present challenges because it can be hard to disentangle effects resulting from antipsychotic treatment versus SCZ pathophysiology [12]. Several functional studies of model organisms or human cells in vitro have focused on specific genes, but few have attempted to capture the genome-wide effects of antipsychotics. Notable exceptions are a bulk RNA sequencing study of haloperidol’s effects in the mouse striatum by Kim et al. [13] and single-cell sequencing analysis of haloperidol and olanzapine effects in medium spiny neurons, astrocytes, and microglia also from mouse striatum [14]. However, neither of these studies focused on clozapine. One prior study used RNA-sequencing to evaluate transcriptional changes due to clozapine in zebrafish after a relatively short (72 h) exposure time [15]. However, full efficacy of antipsychotic therapy is typically considered to take several weeks [16]. Therefore, in the present study, we conduct deep RNA sequencing (RNA-seq) analysis of clozapine-induced differential gene expression in the mouse prefrontal cortex (PFC) following repeated administrations for 21 days. We chose to study the PFC because this brain region has previously been implicated in SCZ and the therapeutic effects of antipsychotics, including clozapine [17]. Tissue was collected following a 24-h washout after the last dose to determine persistent changes resulting from biological remodeling caused by clozapine.

## Materials and Methods

### Subjects

Adult male C57BL/6J mice about eight weeks of age were obtained from the Virginia Commonwealth University (VCU) rodent core laboratories. Mice were housed in groups of 3–5 in standard Plexiglas cages (12 × 18 × 28 cm) in a temperature- and humidity-controlled room (22 ± 2 °C, 50 ± 5%), with food (Teklad 7012 Rodent Diet, Envigo, Madison, WI, USA) and water available ad libitum. All experiments were approved by the Institutional Animal Care and Use Committee of VCU. All procedures were carried out in accordance with the National Research Council’s Guide for Care and Use of Laboratory Animals (2011).

### Drugs and Treatment

**Fig. 1:**
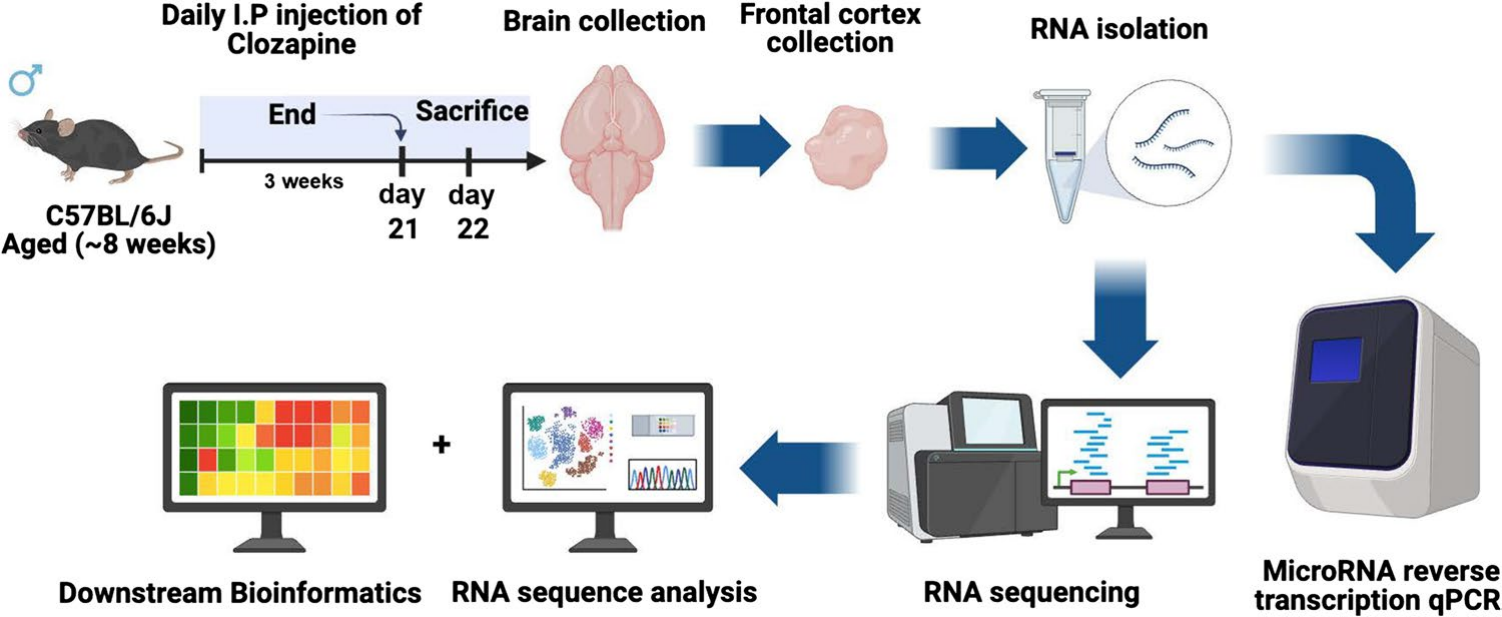
Experimental timeline. Male C57BL/6 mice were subjected to repeated 21 day i.p. clozapine injections. One day after the final injection, mice were sacrificed and the frontal cortex collected for subsequent gene expression analysis. C57BL/6 cartoon from reproduced from DataBase Center for Life Science (DBCLS) submission to Wikimedia Commons under a Creative Commons Attribution 4.0 International license

Clozapine was purchased from Sigma-Aldrich (Burlington, MA, USA). It was first dissolved in 1 M HCl before being prepared in isotonic saline solution for injection, with the pH adjusted to 6.8–7 using 1 M NaOH. For RNA-seq, a dose of 4 mg/kg/day was used based on previous work [18–20] as approximating a low clinical dose in humans. To assess dose-dependent effects on specific RNAs, we also used doses of 1 mg/kg/day and 10 mg/kg/day, in addition to 4 mg/kg/day, to span a tenfold range using specific doses frequently used in prior chronic studies of clozapine in mice [20–22]. Clozapine solution was injected intraperitoneally (i.p.) at 10 ml per kilogram of body weight. Figure 1 shows a schematic representation of the experimental treatments and timeline. Each treatment group (1, 4, and 10 mg/kg or vehicle for 21 days) comprised *n* = 6 subjects.

### Tissue and Nucleic Acid Isolation

Twenty-four hours following the final injection, mice were euthanized via rapid decapitation without anesthesia to prevent interference to CNS gene expression from isoflurane or similar methods. Post-euthanasia, the frontal cortex was swiftly dissected, collected into individual 1.5 ml Eppendorf tubes, and promptly frozen in liquid nitrogen and stored at − 80 °C. Total RNA, including microRNAs, was extracted from approximately 5–10 mg of cortical tissue from each subject using the AllPrep DNA/RNA/miRNA kit (Qiagen, Hilden, Germany). Tissue was homogenized in lysis buffer using a small-bore needle and syringe and extracted according to the manufacturer’s protocol. Nucleic acid concentration and quality were assessed first using a Nanodrop spectrometer (ThermoFisher, Waltham, MA, USA), followed by a BioAnalyzer (Agilent) to yield RNA integrity numbers (RINs).

### RNA Sequencing (RNA-seq)

Cortical RNA samples from subjects receiving either 4 mg/kg/day clozapine for 21 days or vehicle (saline) control were first subjected to poly(A) enrichment using the NEBNext® Poly(A) Magnetic Isolation Module. Poly(A) enrichment robustly captures mRNA and polyadenylated long non-coding RNAs (lncRNAs) [23–27]. Reverse transcription and library preparation for standard, non-stranded Illumina RNA sequencing used the IDT Biosciences RNA library kit, which is optimized for insert sizes of 200–350 bp. Samples were sequenced to a target of > 30 million PE100 clusters each on a single high-output run on a NextSeq2000. Raw data were processed to generate FASTQ files, and FastQC was used to assess base call quality, duplicate reads, adapter contamination, etc. Raw sequence data are available to download from the Sequence Read Archive at the National Center for Biotechnology Information with accession number PRJNA1220692.

### RNA-seq Analysis

Sequencing adapters were removed using TrimGalore v.0.6.4_dev [28]. Quality control was performed using FastQC v0.11.9 (quality base calls, CG content distribution, duplicate levels, complexity level) [29]. Reads were aligned using the STAR v.2.5.2b aligner [30] to the GRCm38/mm10 genome and counted on gene level using featureCounts v2.0.1 [31] using Gencode vM25 gene annotation. RNA-seq counts were preprocessed and analyzed for differential expression using edgeR v.3.30.0 [32]. *P*-values for differentially expressed genes were corrected using False Discovery Rate (FDR) < 5% [33]. Differential exon use analysis was conducted using the DEXSeq package [34]. A flattened version of the Gencode vM25 gene annotation file was created, and.bam files from STAR were processed and analyzed according to the vignette at DOI: 10.18129/B9.bioc.DEXSeq. Specific exons were considered significant if their *p*-values passed FDR < 5%. Using the package *RNASeqPower* [35], we estimate 85% power to detect a twofold change in expression with *n* = 6 per group, coefficient of variation = 0.2, and α = 0.003 (approximately our observed FDR < 5% threshold).

### Downstream Bioinformatics

Pathway analysis of differentially expressed genes used The Gene Ontology Resource (www.geneontology.org) [36–38], release dated 06/17/2024, with reference organism set to *Mus musculus*. A Fisher test was used to test for enrichment with an FDR < 0.05 threshold for declaring significance. To test for enrichment of findings among schizophrenia risk genes, we used the full (*n* = 685 genes) and priority (*n* = 120 genes) gene lists from the 2022 Psychiatric Genomics Consortium paper [39], extended Data Table 1, where genes were selected based on fine mapping of schizophrenia risk loci, and further prioritized based on expression QTL, 3D genomics, and other data. We used the HOM_MouseHumanSequence.rpt data table from Mouse Genome Informatics [40], to map gene identity from mouse to human. Enrichment testing used the unique human genes with identifiable mouse orthologs from this data table (*n* = 19,326) as the background set, and one-sided Fisher exact tests were implemented in R as described previously [41, 42].

### MicroRNA Reverse Transcription qPCR (RT-qPCR)

We assayed microRNAs in the total RNA extracted from subjects receiving three different clozapine doses (1, 4, and 10 mg/kg) or vehicle for 21 days, with tissue collection 24 h after the last dose as shown in Fig. 1. Cortical RNA samples underwent reverse transcription to cDNA using the TaqMan Advanced miRNA cDNA Synthesis Kit (ThermoFisher). Subsequently, each sample was subjected to poly (A) tailing reaction, followed by 5′ adapter ligation, with this adapter serving as the forward-primer binding site for the miR-Amp reaction. MicroRNA qPCR was performed in triplicate for each cDNA template, and a serially diluted (1:2) standard was created using total RNA extracted from untreated C57BL/6 mouse brain. No template controls were also included. TaqMan (ThermoFisher) advanced miRNA assays were as follows: *miR-124-3p* (mmu480901_mir) and *miR-124-5p* (mmu480902_mir). Mouse *U6* (001973, ThermoFisher) was used as the endogenous control. Amplification used TaqMan fast advanced Master Mix on a QuantStudio 3 instrument (Applied Biosystems) with 40 cycles of 20 s at 95 °C, 1 s at 95 °C, and 20 s at 60 °C. Quantification cycle (Cq) values for each sample were determined using the Relative Quantification (RQ) application on the ThermoFisher Cloud. Normalized quantification cycles (ΔCq) were obtained by subtracting the average microRNA Cq from the average *U6* Cq. ΔΔCq was calculated to estimate differences in the treatment groups relative to the vehicle control. The actual fold change was obtained using the formula 2^-ΔΔCq for each drug treatment group separately.

## Results

### RNA-seq analysis of Cortical Gene Expression Following Clozapine Administration

**Fig. 2:**
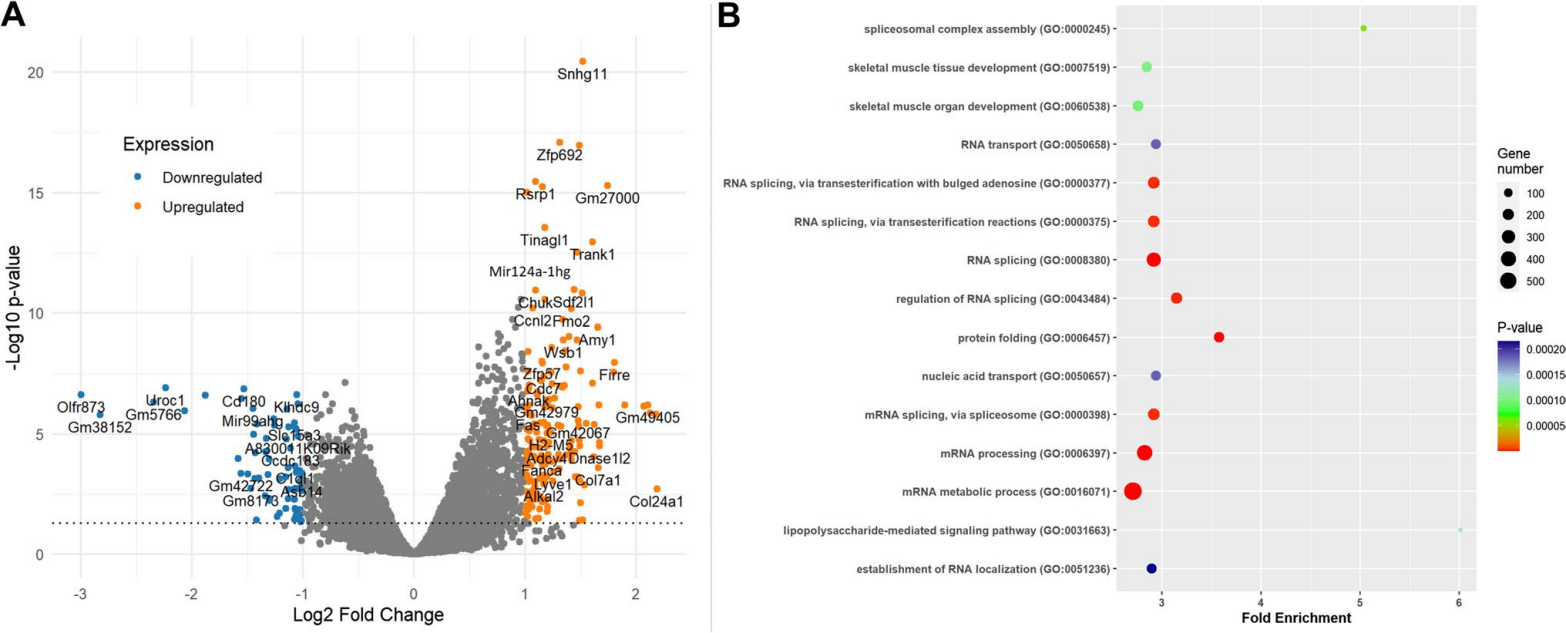
Results from RNA-seq gene expression analysis. (A) Volcano plot showing differentially expressed genes. The x-axis is the log_2_ fold change, while the y-axis is the –log_10_ of the Benjamini–Hochberg adjusted *p*-value of differential expression. The horizontal dotted line shows the FDR < 0.05 threshold for genome-wide significance. Transcripts with *p*-values below this threshold, or those with less than twofold change in expression (up or down) are in gray, while significant transcripts with either > twofold increase in expression (orange) or > twofold reduction in expression (blue) are highlighted. (B) Dot plot of Gene Ontologies (GOs) found to be significantly enriched with genes differentially expressed following repeated clozapine administration (panel A, FDR < 0.05). The size of each dot is proportional to the number of genes in the pathway, while the color is of each dot corresponds to the *p*-value of enrichment (see legend)

Mice received 21 days of repeated administration of clozapine at 4 mg/kg (**Cloz4**) or saline vehicle for the RNA-seq study. Twenty-four hours after the final injection, mice were sacrificed, the frontal cortex dissected, and RNA extracted. All RNA samples had RIN > 7 (range 7.1–8.0). Illumina sequencing was successful with an average of approximately 35.5 million paired-end clusters per sample (range 28 M–39.5 M clusters) of 2 × 100-bp reads. Following alignment to the mm10 reference genome and data processing, principal components analysis (PCA) revealed good separation between cortical RNA profiles for subjects in the Cloz4 and vehicle control groups. Differential expression analysis of Cloz4 and vehicle groups revealed 1037 differentially expressed genes (DEGs) in the PFC at FDR < 0.05 (Fig. 2A) out of > 16 K transcripts detected above quality control thresholds. Among the significant findings, most transcripts (*n* = 813, 78.5%) were protein-coding, while *n* = 98 (9.4%) were annotated as lncRNAs, with the remainder being mostly pseudogene transcripts. As can be seen in Fig. 2A, a larger number of significant DEGs were upregulated (*n* = 661 total; *n* = 146 where log_2_FC > 1) as compared to downregulated (*n* = 376 total; *n* = 49 where log_2_FC < −1). The complete results are provided in Supplementary Table 1.

To determine if DEGs showed consistent biological themes, we ran pathway analysis on the significant findings (FDR < 0.05), and the results for Gene Ontology (GO) Biological Processes are shown in Fig. 2B, sorted in descending order of fold enrichment. We show the top 15 significant GOs in Fig. 2B**,** and the complete set of GO findings are provided in Supplementary Table 2. Several of the top GOs enriched with our DEGs were involved in RNA processing; most notable (by fold enrichment) were spliceosomal complex assembly (GO:0000245), regulation of RNA splicing (GO:0043484) and RNA transport (GO:0050658). This result implies that repeated clozapine administration is affecting the expression of many cortical genes involved in the regulation of expression itself, including RNA splicing.

### Non-coding RNAs Showing Differential Cortical Expression in Response to Clozapine

As described above, a relatively large number (*n* = 98) of lncRNAs were detected to be differentially expressed in the cortex of clozapine-treated subjects. Of these 98 genes, exactly half (*n* = 49) showed |log_2_FC|> 1. The top 10 lncRNA findings |log_2_FC|> 1 are shown in Table 1. The most significant lncRNA overall was *Snhg11*, the small nucleolar host gene 11, which showed an almost threefold (2.87 ×) increase in expression in the clozapine-treated subjects. Recently, *Snhg11* was implicated in synaptic plasticity and adult neurogenesis in the hippocampus [43], but little else is known about its function in the brain. The second most significant lncRNA is *Gm27000*, which is annotated as a predicted transcript and consequently is uncharacterized. However, the third most significant lncRNA was *Mir124a-1hg*, the microRNA 124–1 host gene. *Mir124a-1hg*, previously named *Rncr3*, is the dominant source of microRNA 124 [44], a well-characterized microRNA that is abundantly expressed in the brain [45]. Most relevant to the current project, it regulates cortical dopaminergic signaling [46] and was recently implicated in mediating the polygenic risk for psychosis via glutamate signaling [47]. Given the potential relevance of miR-124 to antipsychotic drug action, we decided to further investigate this finding in the context of clozapine.

**Table 1:** Top 10 most significant long non-coding RNAs (lncRNAs) showing differential expression (|log_2_FC|> 1) in mouse cortex following repeated (21 day) clozapine administration at 4 mg/kg/day. Ensembl gene accession numbers are provided in addition to the standard gene symbol, logFC is log_2_(fold change), p-val is the *p*-value of association in RNA-seq analysis, FDR is the False Discovery Rate

ENSMBL ID	logFC	*p*-value	FDR	gene symbol	biotype
ENSMUSG00000044349	1.52	3.67E-21	6.19E-17	*Snhg11*	lncRNA
ENSMUSG00000097961	1.74	5.26E-16	1.67E-12	*Gm27000*	lncRNA
ENSMUSG00000097545	1.18	2.85E-11	3.21E-08	*Mir124a-1hg*	lncRNA
ENSMUSG00000097391	1.24	2.72E-09	1.48E-06	*Mirg*	lncRNA
ENSMUSG00000085396	1.80	1.12E-08	4.41E-06	*Firre*	lncRNA
ENSMUSG00000092274	1.37	1.68E-08	6.11E-06	*Neat1*	lncRNA
ENSMUSG00000117768	1.61	7.38E-08	1.97E-05	*8030456M14Rik*	lncRNA
ENSMUSG00000084904	1.31	1.03E-07	2.41E-05	*Gm14827*	lncRNA
ENSMUSG00000097277	1.27	3.32E-07	6.22E-05	*2900076A07Rik*	lncRNA
ENSMUSG00000085609	1.20	6.05E-07	9.45E-05	*1700016P03Rik*	lncRNA

**Fig. 3:**
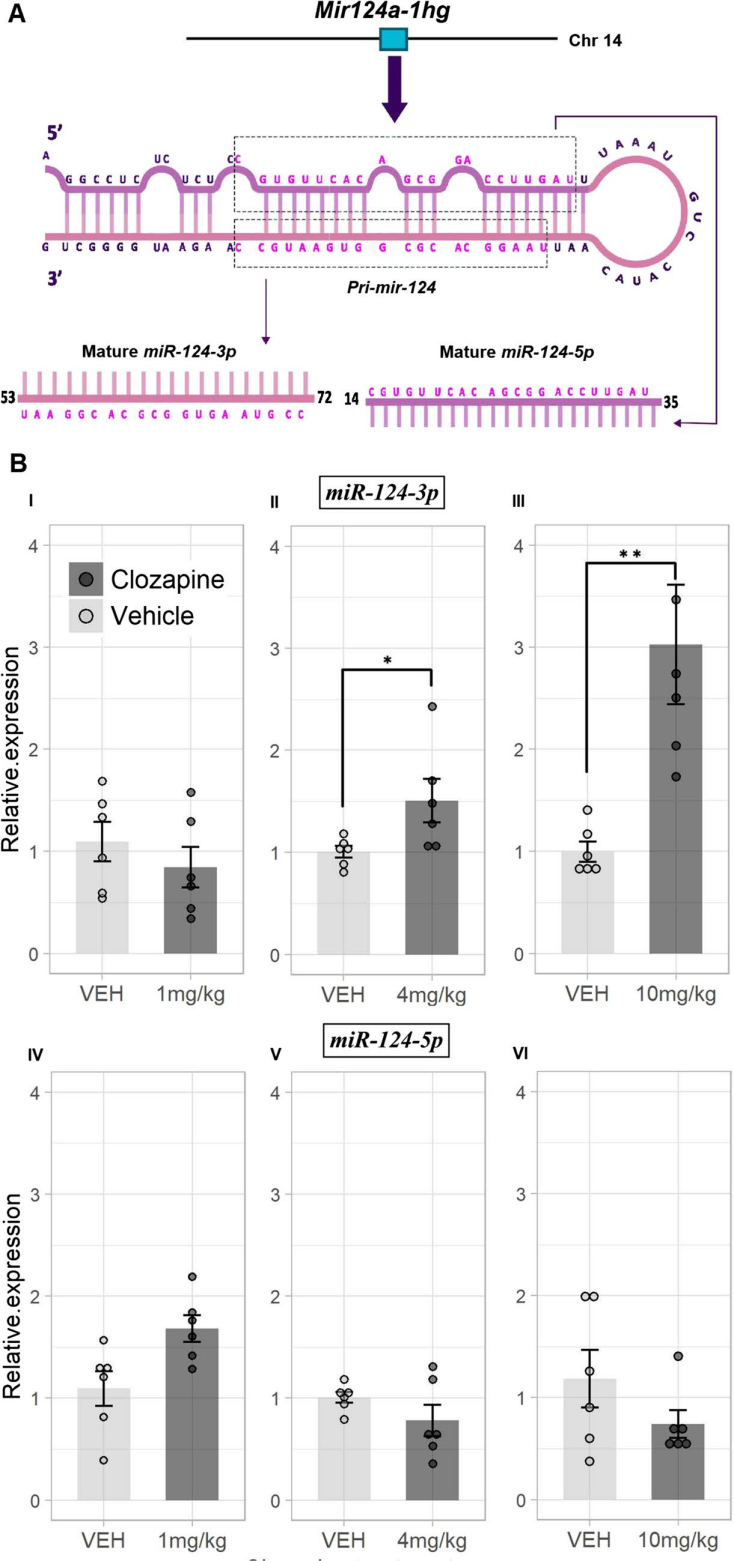
Dose-dependent response of cortical *miR-124-3p* to repeated clozapine administration for 21 days. Panel (A) shows the microRNA 124 host gene (*Mir124a-1hg*) that was significantly up-regulated in our RNA-seq analysis of the cortex following clozapine administration at 4 mg/kg for 21 days. *Mir124a-1hg* is spliced to yield the *Pri-mir-124* hairpin, which is the further processed to yield the mature *miR-124-3p* and *miR-124-5p* microRNAs. Data from miRbase [48]. Panel (B) shows the results of qPCR analysis of the two mature microRNAs in the cortex of subjects exposed to three different clozapine doses of 1, 4, and 10 mg/kg/day respectively for 21 days. The upper set of three barplots (1, II and III) show the results for *miR-124-3p* while the lower set of three (IV, V and VI) show the results for *miR-124-5p.* For each plot, the x-axis shows the clozapine dose and vehicle groups, while the y-axis shows the normalized fold change (2^−(ΔΔCq)^) relative to the control (vehicle, VEH) group. * = *p* < 0.05, ** = *p* < 0.01

*Mir124a-1hg*, which is located on chr14qD1, contains the miR-124 stem loop hairpin *Mir124a-1* (mmu-mir-124–1) in its fourth exon. The *Mir124a-1* hairpin is further processed to yield the mature *miR-124-3p* and *miR-124-5p* microRNAs [48] (see Fig. 3A). There are two other miR-124 hairpin genes in mouse. *Mir124a-2* (*mmu-mir-124–2*) is located within *Mir124-2hg* on chr3qA1. Notably, *Mir124-2hg* was also slightly up-regulated in our data (1.45-fold increase, FDR < 0.2). A third miR-124 hairpin in mouse is *Mir124a-3* (*mmu-mir-124–3*) on chr2qH4 [49]. However, *Mir124a-3* does not have an annotated host gene in the mouse reference genome (mm10), and the hairpin genes themselves are too small to be captured by our RNA-seq approach, which is optimized to capture fragments 200–350 bp. Nevertheless, our significant finding of *Mir124-1hg* upregulation, in addition to the weaker *Mir124-2hg* upregulation, suggested that further analysis of the mature *miR-124-3p* and *miR-124-5p* forms should be studied in the context of clozapine. We performed qPCR analysis of *miR-124-3p* and *miR-124-5p* levels in mouse cortical RNA following 21-day exposure to 1 mg, 4 mg or 10 mg daily clozapine injections. As shown in Fig. 3B, we observed a significant up-regulation of cortical *miR-124-3p* in response to clozapine, but no significant change with *miR-124-5p* at any dose. More specifically, the *miR-124-3p* upregulation appeared to be dose-dependent, with no significant change at 1 mg/kg/day, a significant (*p* < 0.05) 1.6-fold increase at 4 mg/kg/day, and an almost threefold increase at 10 mg/kg/day. Overall, these results indicate up-regulation of *miR-124-3p* signaling in response to repeated clozapine administration.

### Differential RNA Splicing in the Cortex Induced by Repeated Clozapine Administration

**Fig. 4:**
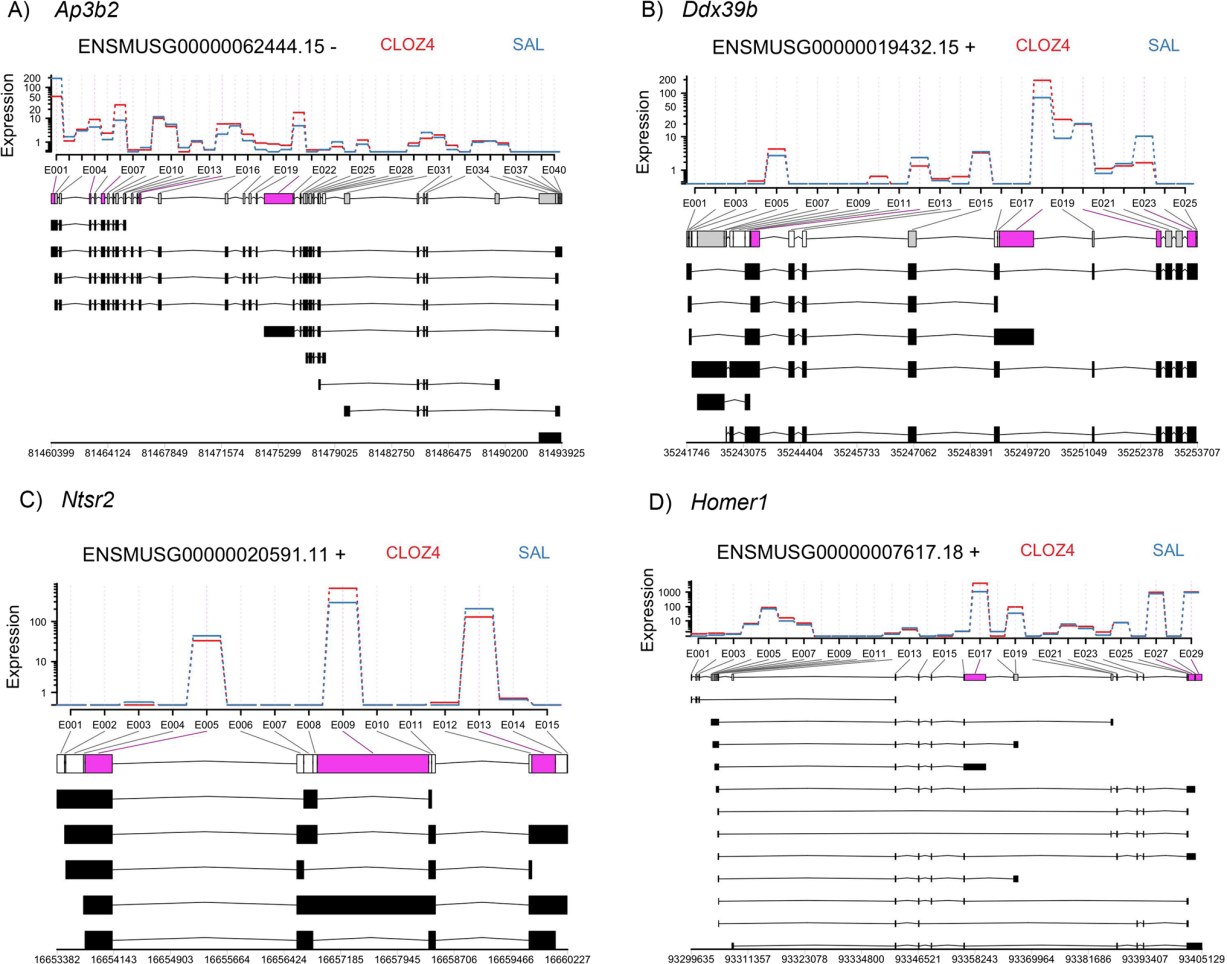
Plots showing differential exon use in the cortex following 4 mg/kg/day clozapine for 21 days. The exon structure of each of the four most significant genes are shown, with the relative abundance of each exon transcript in clozapine (CLOZ4, red) and vehicle (SAL, blue) plotted. Exons showing significant differences in abundance are colored in magenta. Genomic positions are for mouse genome build mm10. Plots were made using the DEXSeq package

Our RNA-seq analysis revealed several DEGs involved in RNA splicing and processing, as shown in the Gene Ontology analysis in Fig. 2B. This result led us to hypothesize that clozapine would alter splicing patterns. To test this, we re-analyzed our RNA-seq data to identify differential exon use (DEU) using the DEXSeq package. Overall, we found 1925 exons across the genome showing significantly different patterns of expression (FDR < 0.05) following clozapine administration at 4 mg/kg/day, and these exons mapped to 1440 unique genes. Of the 1925 differentially expressed exons, *n* = 1167 had |log_2_FC|> 1. These results are provided in Supplementary Table 3. For illustrative purposes, the DEU plots for the top four genes (*Ap3b2*, *Ddx39b*, *Ntsr2* and *Homer1*) are shown in Fig. 4. Each of these genes had more than one significant differentially expressed exon, which are shown in purple in the figure.

To gain further insight into the overlap between clozapine’s effects and SCZ etiology, we conducted an integrative analysis of our results with the most recent, published, genome-wide association study (GWAS) findings from the Psychiatric Genomics Consortium (PGC) [39]. In Table 2, we first show the overlap between our clozapine DEGs and SCZ risk genes from GWAS. Then, we show the overlap between our genes showing differential exon use and SCZ risk genes from GWAS. We observed enrichment of our results from both categories among SCZ risk genes. There were 29 genes from the full list of PGC SCZ risk genes whose orthologs showed differential expression in the mouse cortex as a result of repeated clozapine administration, and 50 genes from the full list of SCZ risk genes whose orthologs showed differential exon use. To better understand the roles of these SCZ risk genes affected by clozapine, we performed GO analysis. The 29 SCZ risk genes showing differential expression following clozapine administration were not enriched in any ontologies. However, the 50 genes showing differential exon use were highly enriched in the Cellular Component categories “apical dendrite” and “distal axon” (Table 2). This result indicates that clozapine is affecting the expression of SCZ risk genes that localize to these subcellular regions.

**Table 2:** Data integration and pathways. The table is divided into three sections for each dataset under investigation. First, “Clozapine genes” shows the integration of genes showing differential expression in our RNA-seq study (Fig. 2) with all SCZ risk genes (*n* = 685) and priority SCZ risk genes (*n* = 120) from the Psychiatric Genomics Consortium SCZ GWAS by Trubetskoy et al. (2022). Next, “Clozapine alt exon genes” shows the same analysis with the genes showing differential exon use in our RNA-seq study. The lower portion of the table shows the results of Gene Ontology (GO) analysis of the 50 SCZ risk genes showing differential exon use in our study (bold). All GO categories were significant FDR < 0.05. (The 29 SCZ risk genes showing differential expression in our study were not significantly enriched in any GO category.)

	set size (N genes)	Overlap	OR enrich	*p*-value
**Clozapine genes**				
All SCZ risk genes	685	29	1.58	0.01677
Priority SCZ risk genes	120	10	2.56	0.009254
**Clozapine alt exon genes**				
All SCZ risk genes	685	**50**	1.65	0.00117
Priority SCZ risk genes	120	9	1.3	0.2745
**GO analysis of 50 SCZ risk genes showing differential exon use**				
	set size (N genes)	Overlap	Fold enrich	*p*-val
Apical dendrite	17	3	74.12	8.43E-06
Distal axon	274	6	9.2	4.56E-05
Axon	651	8	5.16	1.38E-04

## Discussion

In this study, we have shown extensive changes to genomic regulation following repeated clozapine administration, as evidenced by changes in the expression levels of several coding and non-coding RNAs. Moreover, we demonstrated that repeated clozapine administration affects expression of genes involved in regulation of splicing, which is accompanied by extensive changes to exon use patterns implying the use of alternative mRNA isoforms. Many of the genes differentially regulated by clozapine in our study are also SCZ risk genes, and these are particularly enriched among synaptic genes localized to apical dendrites and axons.

SCZ is now well established to be a highly heritable, complex disorder [50], with hundreds of risk genes now discovered [39]. Transcriptional change and disruptions to isoform use are well-known features of SCZ [51, 52], but the genomic mechanisms of antipsychotic drugs and how these may modify the expression of SCZ risk genes are less well understood. In the last decade, epigenetic mechanisms have been established as important for antipsychotic response, particularly in relation to histone deacetylase enzymes and the regulation of metabotropic glutamate receptors [8, 19, 20, 53]. On the other hand, genome-wide studies of epigenetic and/or expression effects of antipsychotics in the brain are few. One such study is that of Abrantes et al. (2022) [14], who studied the effects of chronic olanzapine and haloperidol administration on gene expression in mouse striatum using single-cell RNA-seq. Similar to the current study, they found transcriptional splicing to be affected, in addition to enrichment of SCZ risk genes among their findings. Kim et al. (2018) [13], also showed significant overlap between SCZ risk genes and differentially expressed genes from bulk RNA-seq of mouse striatum following chronic haloperidol administration. Despite the drugs and brain regions differing between these studies and ours, there is a common theme of altered splicing and altered expression of SCZ risk genes following antipsychotic administration. Taken together, these findings suggest that further insight could be derived from more systematic studies of several antipsychotics and brain regions, to look for specific convergences of genes and mechanisms.

We observed differential exon use (DEU), implying altered splicing, at fifty SCZ risk genes in the cortex following repeated clozapine administration. These genes were significantly enriched in “apical dendrite” and “distal axon” gene ontologies. The “apical dendrite” genes matching DEU genes in our data were *Elavl4*, *Ptk2b* and *Slc4a10*. Of these *Ptk2b* is a predicted target of *miR-124-3p* [54] and shows reduced expression of one exon (log_2_FC = − 1.38) in our data. The “distal axon” overlapping set included all three of the “apical dendrite” genes in addition to *Ap3d1*, *Klc1* and *Mapt*. *Ap3d1* and *Mapt* are also predicted targets of *miR-124-3p* [54] and each shows reduced expression of one exon (log_2_FC = − 3.07, − 3.08, respectively) in our data. The fact that these *miR-124-3p* targets are showing reduced expression in our data is consistent with the role of microRNAs as silencers of expression. The specific localization of these differential effects may be of relevance for SCZ. Apical dendrites are a feature of pyramidal neurons and extend toward the surface of the cortex, as opposed to the basal dendrites that radiate away from the cell body in several directions [55]. Dendritic spine loss, particularly in layer III of the cortex, has been shown in postmortem analysis of brain tissue from SCZ patients [56]. Apical dendrites specifically have been reported to be ~ 70% reduced in patients with SCZ, but this study was in relatively small numbers [57]. Kathuria et al. (2023) [58] recently analyzed cortical neurons differentiated from induced pluripotent stem cells (iPSC) from SCZ patients and controls. They showed significant reduction in dendritic spine density for neurons expressing a layer III marker (CUX1), mirroring the prior clinical findings. Analysis of transcriptomic data from the same cells identified alternative splicing at neurexin 3 (*NRXN3*) as a key driver of the dendritic spine loss. Most relevant to the current study, the splicing change at *NRXN3* and the dendritic spine pathology noted by Kathuria et al. were reversed by clozapine [58]. *NRXN*3 was among the genes we found to show significant differential exon use in response to clozapine (ENSMUSG00000066392, Supplementary Table 3). *NRXN3* is also a predicted target of *miR-124-3p* [54]. A small number of other studies have shown that clozapine affects apical dendrites. For example, Willins et al. (1999) showed that clozapine reduced the numbers of serotonin 2 A (5HT2A) receptors on apical dendrites in rats treated with clozapine for seven days [59]. Pyramidal neurons and dendritic spines are considered likely to play a key role in SCZ pathophysiology and treatment response [60, 61] and our findings integrate with these aspects of the disorder.

We found that both lncRNAs and the microRNA *mir-124-3p* to be differentially expressed following clozapine administration. Noncoding RNAs have been implicated in SCZ etiology and treatment response [62]. However, lncRNA characterization and annotation is lacking relative to protein-coding RNAs, limiting the efficacy of pathway analysis to determine biological themes among these findings. Nevertheless, several of our top genes are of interest, such as *Snhg11,* which is implicated in synaptic plasticity and adult neurogenesis [43]. Arguably our most exciting finding, however, was *Mir124a-1hg*. This lncRNA is the dominant source of microRNA 124 (*miR-124*) [44], one of the most abundant microRNAs in the brain [45] that is estimated to account for between one-quarter to one-half of the total brain microRNA content [63]. MicroRNA 124 is implicated in the regulation of neurogenesis [64], neural stem cell proliferation and differentiation [65], dendritic morphogenesis, and spine density [66] and synaptic plasticity [67]. Interestingly, it has also been implicated in regulation of mRNA splicing in neurons [68]. Most relevant to the current project, mice deficient for *miR-124* exhibit deficits in the sensory gating measure of prepulse inhibition (PPI), altered response to psychostimulants, social deficits and enhanced cortical expression of dopamine receptor D2 (DRD2) [46]. These effects align with traditional rodent models of psychosis-like deficits used for antipsychotic drug discovery, e.g. PPI deficits in rodents are reversed by drugs showing antipsychotic efficacy, and DRD2 is the classical antipsychotic drug target receptor. Furthermore, *miR-124-3p* was specifically implicated in mediating the polygenic risk for psychosis [47] and as a regulator of other SCZ risk genes [69–71]. The human microRNA 124 host gene (*MIR-124_1HG*) was significantly downregulated in two of the largest gene expression studies of SCZ undertaken by the PsychENCODE Consortium. Specifically, *MIR-124_1HG*, alias LINC00599 (see Entrez Gene ID:157,627), was significantly (FDR = 8.0E-3) downregulated in bulk RNA-seq of postmortem cortical tissue from SCZ patients in PsychENCODE phase 1 [52], in addition to being significantly downregulated in several excitatory neuron subtypes in single nucleus gene expression analysis of postmortem cortical tissue from SCZ patients conducted during PsychENCODE phase 2 [72]. The most significant (FDR = 7.6E-05) downregulation was in layer 2 excitatory neurons (Ex-L2). Finally, the *MIR-124_1HG* locus has been associated with both SCZ and bipolar disorder in large-scale GWAS [39, 73]. Taken together, these findings suggest a role for miR-124 and its host gene in antipsychotic response and SCZ, but more research is needed to determine how miR-124 responds to other antipsychotics and CNS drug classes.

Until the recent approval of xanomeline-trospium chloride, which is a muscarinic agonist [74], all antipsychotics targeted dopamine receptors, and are antagonists at DRD2 in particular. For many decades, SCZ drug development was incremental. Arguably one reason for the lack of rapid advance is the lack of preclinical markers of antipsychotic action. If we consider antidepressant drug discovery, upregulation of brain-derived neurotrophic factor (BDNF) signaling has been shown to be a key component of antidepressant response, through stimulating increased synaptic plasticity in the hippocampus and cortex [75]. Notably, ketamine also up-regulates BDNF on a more rapid timeline than conventional antidepressants, in line with its more rapid, atypical mode of action [76]. Thus, BDNF functions as a preclinical marker of antidepressant response in the CNS. No marker with similar properties is available for antipsychotics, to the best of our knowledge. However, our results suggest that specific microRNAs could, with further research, fill this gap. MicroRNAs could also serve as clinical biomarkers of antipsychotic response, whether from lymphocytes or from CNS neuron-derived exosomes in blood [77, 78].

There are some limitations to our study design. First, our findings will require replication in independent samples. Second, we studied only one antipsychotic drug, clozapine. Although there are strong reasons to choose clozapine, it will be necessary to verify if the gene expression findings here are specific to clozapine or common to other antipsychotics. Third, we only used male mice, which limits the generalizability of the findings, as females may exhibit different responses to clozapine. Fourth, the study focuses solely on the prefrontal cortex, and while this brain region is highly relevant for investigating the effects of antipsychotic drugs, clozapine may have varying effects on gene expression in other brain regions. Another potential limitation is our use of oligo(dT) capture in RNA-seq. This method captures many lncRNAs [23] that are transcribed by RNA polymerase II and, therefore, share similar features to mRNAs, such as a poly(A) tail [24–27]. The lncRNAs that we highlighted (e.g. *Snhg11*, *Mir124a-1hg*) have polyadenylation signals, as indicated by the PolyASite database [79], so they would be captured by our sequencing method. However, many lncRNAs are not polyadenylated, so a more comprehensive method would be ribosomal RNA depletion. Finally, we did not include behavioral or functional assessments that could provide mechanistic insight into the physiological relevance of *miR-124-3p* expression for antipsychotic response, which will require further study.

## Supplementary Information

Below is the link to the electronic supplementary material.

Supplementary Table 1 (CSV 2.18 MB)

Supplementary Table 2 (XLSX 14.7 KB)

Supplementary Table 3 (XLSX 446 KB)

## Data Availability

Sequence data are available to download from the Sequence Read Archive at the National Center for Biotechnology Information with accession number PRJNA1220692.
